# SLAF-seq: An Efficient Method of Large-Scale *De Novo* SNP Discovery and Genotyping Using High-Throughput Sequencing

**DOI:** 10.1371/journal.pone.0058700

**Published:** 2013-03-19

**Authors:** Xiaowen Sun, Dongyuan Liu, Xiaofeng Zhang, Wenbin Li, Hui Liu, Weiguo Hong, Chuanbei Jiang, Ning Guan, Chouxian Ma, Huaping Zeng, Chunhua Xu, Jun Song, Long Huang, Chunmei Wang, Junjie Shi, Rui Wang, Xianhu Zheng, Cuiyun Lu, Xiaowu Wang, Hongkun Zheng

**Affiliations:** 1 Heilongjiang River Fisheries Research Institute, Chinese Academy of Fishery Sciences, Harbin, China; 2 Biomarker Technologies Corporation, Beijing, China; 3 Key Laboratory of Soybean Biology in Chinese Ministry of Education, Northeast Agricultural University, Harbin, China; 4 Institute of Vegetables and Flowers, Chinese Academy of Agricultural Sciences, Beijing, China; Leuven University, Belgium

## Abstract

Large-scale genotyping plays an important role in genetic association studies. It has provided new opportunities for gene discovery, especially when combined with high-throughput sequencing technologies. Here, we report an efficient solution for large-scale genotyping. We call it specific-locus amplified fragment sequencing (SLAF-seq). SLAF-seq technology has several distinguishing characteristics: i) deep sequencing to ensure genotyping accuracy; ii) reduced representation strategy to reduce sequencing costs; iii) pre-designed reduced representation scheme to optimize marker efficiency; and iv) double barcode system for large populations. In this study, we tested the efficiency of SLAF-seq on rice and soybean data. Both sets of results showed strong consistency between predicted and practical SLAFs and considerable genotyping accuracy. We also report the highest density genetic map yet created for any organism without a reference genome sequence, common carp in this case, using SLAF-seq data. We detected 50,530 high-quality SLAFs with 13,291 SNPs genotyped in 211 individual carp. The genetic map contained 5,885 markers with 0.68 cM intervals on average. A comparative genomics study between common carp genetic map and zebrafish genome sequence map showed high-quality SLAF-seq genotyping results. SLAF-seq provides a high-resolution strategy for large-scale genotyping and can be generally applicable to various species and populations.

## Introduction

The development of genotyping technologies has been synchronous with the development of molecular markers. Traditional gel-based molecular markers have played important roles in genotyping assays during the past 20 years [1]–[3]. Most of these have been based on length polymorphisms. However, restrictions in throughput have rendered large-scale genotyping difficult.

The collection of genome sequences and discovery of thousands of single nucleotide polymorphisms (SNPs) have made large-scale microarray-based SNP genotyping possible [4]–[6]. Microarray-based genotyping has been used in many well-studied species [7]–[10]. Millions of pre-discovered SNPs can be used to design probes, and sequence variations between samples can be detected by hybridization. However, microarray quality relies on the SNP profiling quality of the species. The more known SNPs there are, the higher the microarray quality. The limited number of known SNPs limits the applicability of this technology in many cases.

High-throughput sequencing technologies can provide new strategies for sequence-based SNP genotyping. Whole-genome resequencing strategies can be used to genotype a large number of variations among samples [11]–[14]. Population size may range from 8 to 50. This strategy mainly focuses on SNP discovery and evolutionary studies on domestication. For large populations, whole-genome deep sequencing is still cost-prohibitive. However, population size greatly affects the accuracy of association studies. To reduce the costs of sequencing, two kinds of strategies have been developed within the past several years. One of these strategies involves reducing sequence depth. Low-depth whole-genome resequencing methods have mainly been used in the mapping of populations. Huang et al. genotyped a rice RILs with 0.02-fold coverage per line [15].A slide-window approach was developed to improve SNP accuracy. Xie et al. sequenced 238 RILs with 0.055-fold genome coverage. This showed some improvement in parent's sequence independence, which reduced the cost of sequencing the parents' genomes [16]. However, it relies on high-quality reference genome sequences, and the sequence library cost cannot be reduced when processing large populations. The other strategy involves reducing the complexity of the genome with reduced representation library (RRL) sequencing [17]–[20]. Many properties can be used to prepare reduced representations. One of the simplest methods is the separation and purification of restriction fragments within a given size range. The use of RRL for SNP discovery was first described using Sanger sequencing during the Human SNP Map Project [17], [21]. In recent studies, RRL were used with high-throughput sequence technologies. Baird et al. genotyped a stickleback F2 population, and a barcode was developed to distinguish each individual [22]. The restriction-site-associated fragment end was sequenced using an Illumina single-end protocol. Hyten *et al.* performed RRL sequencing on a wild soybean strain for SNP discovery [18]. Tassell *et al.* studied 66 cattle representing three populations [20]. These studies based on RRL sequencing strategies all relied on the reference genome sequences. Single-end sequencing was pursued, but this limits the study in many ways and is somewhat prone to error.

Here we developed a strategy for the *de novo* SNP discovery and genotyping of large populations using an enhanced RRL sequencing method. Reference genome sequences and polymorphism information are not necessary when this strategy is used. With barcode multiplexed sequencing, large populations with large numbers of loci can be genotyped simultaneously. With pre-designed RRL schemes, repetitive sequences can be avoided, and the selected fragment number can be decided for personalized research purposes to maintain the balance between marker density and population size. In this study, we genotyped a common carp (*Cyprinuscarpio L.*) F_1_ population. The genetic map that we constructed had the highest density of any map made of any organism lacking reference genome sequences to date. A weight-related QTL was located using genome-wide association studies. The quality of the genotyping process was validated. This strategy is suitable for many types of populations and species.

## Materials and Methods

### Ethics statement

All experimental procedures were conducted in conformity with institutional guidelines for the care and use of laboratory animals in Centre for Applied Aquatic Genomics of the Chinese Academy of Fishery Sciences. The protocol was approved by the Committee on the Ethics of Animal Experiments of the Centre for Applied Aquatic Genomics at Chinese Academy of Fishery Sciences (2011AA1004020012).

### SLAF library construction and high-throughput sequencing

**Figure 1:**
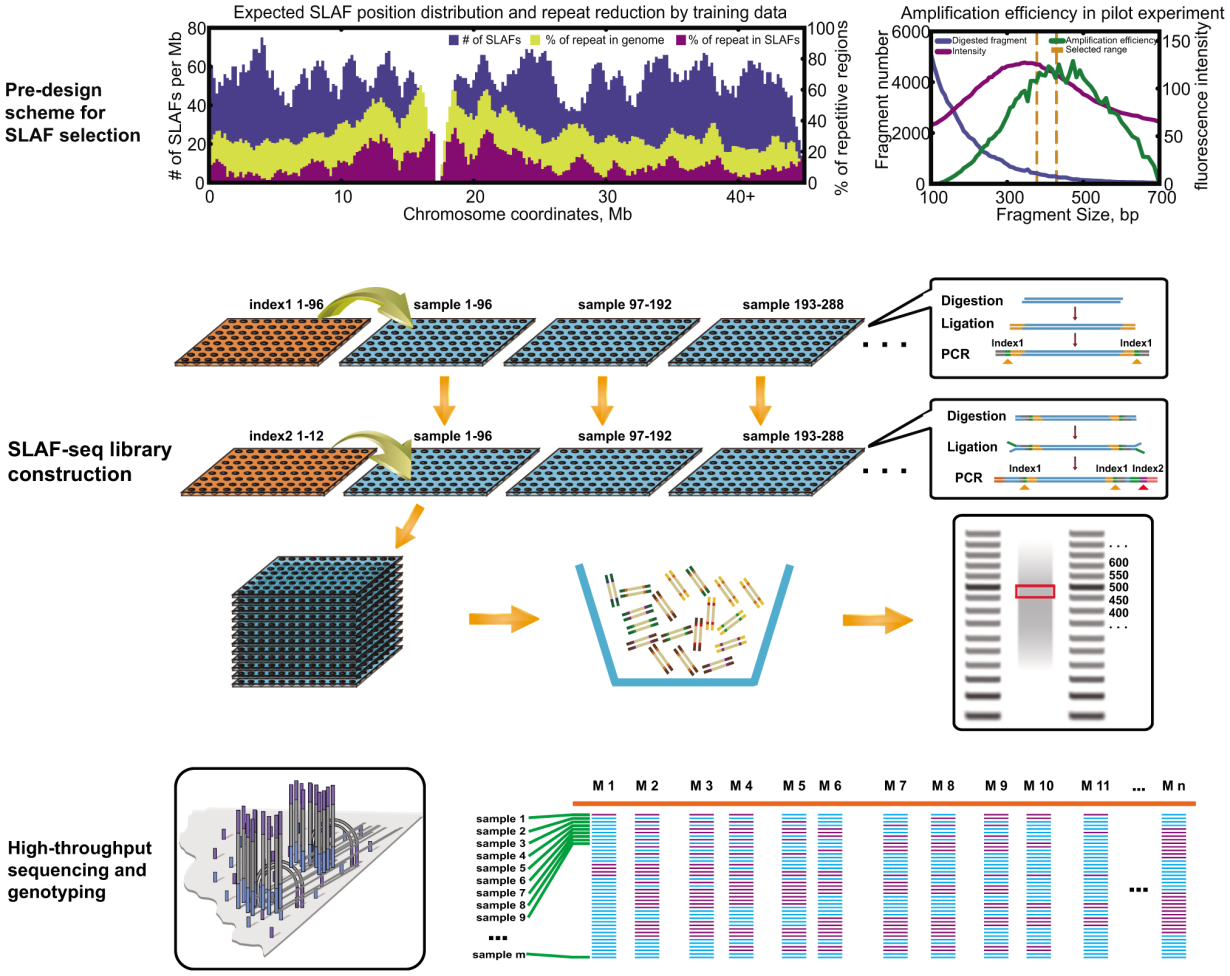
SLAF-seq flowchart. i) Pre-design scheme for SLAF selection using training data. The reduced representation design must be decided based on marker efficiency characteristics, which include random distribution throughout the genome, uniqueness in the genome, and consistent amplification efficiency among selected markers. A pilot experiment was performed to evaluate the amplification efficiency based on the pre-designed scheme. ii) SLAF-seq library construction. Genomic DNA was digested by groups of enzymesdesigned for individuals. Double barcodes were added to two round PCR reactions to discriminate each individual and to facilitate the pooling of samples for size selection, which maintained consistent fragment size among individuals. iii) Deep sequencing for the pooled RRLs with the Illumina paired-end sequencing protocol, and genotype definition and validation by software.

Specific-locus amplified fragment sequencing (SLAF-seq) is an efficient method of large-scale genotyping, which is based on RRL and high-throughput sequencing. The procedure is shown in Figure 1. First, we performed a SLAF pre-design experiment. The enzymes and sizes of restriction fragments were evaluated using training data. Three criteria were considered: i) The number of SLAFs must be suitable for the specific needs of the research project. ii) The SLAFs must be evenly distributed through the sequences to be examined. iii) Repeated SLAFs must be avoided. These considerations improved the efficiency of SLAF-seq. To maintain the sequence depth uniformity of different fragments, a tight length range was selected (about 30∼50 bp) and a pilot PCR amplification was performed to check the RRL features in this target length range, which would ordinarily include fragments with similar amplification features on the gel. When non-specific amplified bands appeared on the gel, then we will repeat the pre-design step to produce a new scheme.

Next, we constructed the SLAF library in accordance using the pre-designed scheme. For the common carp F1 population, genomic DNA was incubated at 37°C with *Mse*I (New England Biolabs, NEB), T4 DNA ligase (NEB), ATP (NEB),and *Mse*I adapter. Restriction-ligation reactions were heat-inactivated at 65°C, and then digested for additional restriction enzyme *Alu*I at 37°C. The PCR reaction was performed using diluted restriction-ligation samples, dNTP, Taq DNA polymerase (NEB) and *Mse*I-primer containing barcode1. The PCR productions were purified using E.Z.N.A.® Cycle Pure Kit (Omega) and pooled. The pooled sample was incubated at 37°C with *Mse*I, T4 DNA ligase, ATP and Solexa adapter. The sample was purified using a Quick Spin column (Qiagen), then run out on a 2% agarose gel. Fragments with 450∼500 bp (with indexes and adaptors) in size were isolated using a Gel Extraction Kit (Qiagen). These fragment products were then subjected to PCR amplification with Phusion Master Mix (NEB) and Solexa Amplification primer mix to add barcode2. Phusion PCR settings were as listed in the Illumina sample preparation guide. Samples were gel purified, excising DNA 450∼500 bp, which was diluted for sequencing.

Then, pair-end sequencing was performed upon the selected SLAFs using an Illumina high-throughput sequencing platform (Illumina, Inc; San Diego, CA, U.S.). SNP genotyping and evaluation were then performed.

### SLAF-seq data grouping and genotype definition

Software was developed to deal with SLAF-seq data. Procedures are shown in Figure S1. All SLAF pair-end reads with clear index information were clustered based on sequence similarity. To reduce computing requirements, identical reads were merged together, and sequence similarity was detected using one-to-one alignment by BLAT [23] (-tileSize = 10 -stepSize = 5). Sequences with over 90% identity were grouped in one SLAF locus.

Alleles were defined in each SLAF using the MAF evaluation. To prevent false positive results, the sequence error rate was estimated using the rice data as a control. These were obtained using the same sequencing scheme as that used with common carp (Figure S1B). True genotypes had markedly higher MAF values than genotypes containing sequence errors. Tags with sequence errors were corrected to the most similar genotype to improve data efficiency. In mapping populations of diploid species, one locus can contain at most 4 genotypes, so the groups containing more than 4 tags were filtered out as repetitive SLAFs. SLAFs with sequence depth less than 213 were defined as low-depth SLAFs and were filtered out of the following analysis. Only groups with suitable depth and fewer than 4 seed tags were identified as high-quality SLAFs, and SLAFs with 2–4 tags were identified as polymorphic SLAFs.

To evaluate the accuracy of our genotyping objectively, a Bayesian approach was proposed. Using the coverage of each allele and the number of single-nucleotide polymorphism, we calculated a posteriori conditional probability that a given individual would have a specific genotype at a corresponding locus. We proceeded as follows. Supposing there were 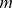 alleles at any given locus, denoted as 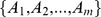. For a diploid species, the number of all possible genotypes was equal to 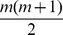 and 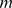 is less than five regardless of the type of segregation of the loci. We assign a priori probability to each genotype according to the theoretical frequencies with which these genotypes would occur in such a finite probability space. For a homozygous genotype, this priori probability would equal 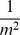, but it would be double that for a heterozygous genotype. Consider a pair of distinguished alleles 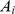 and 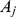, the probability of sequencing one allele to another can be calculated using the following formula:
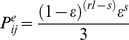
(1)Here is the average ratio of sequencing error. In our model it took on a value of 0.015 for the Illumina sequencing platform, and we used 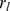 to represent the length of reads and 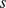 for number of single-nucleotide polymorphisms. Based on this, we obtained the probability of allele 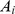 conditioned on the genotype.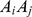, denoted as 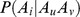. The depth observation of allele 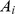 was assumed to be 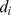, and the conditional probability of observation 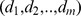 of each genotype can be illustrated as follows:
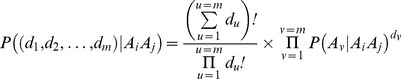
In this way, we determined the probability of assigned genotype conditioned on the following coverage observation:
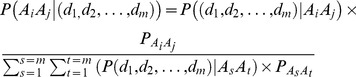
The probability was translated to a genotyping quality score finally using:
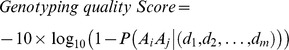
The final genotyping quality score value indicated the confidence with which the genotype had been called. In particular, when the difference in depth between both alleles exceeded 1∶5, the score value could be modified directly using formula (1) due to systematic bias. The upper bound of the score is 30.

This genotyping quality score was used to select qualified markers and individuals for subsequent analysis. This was a dynamic optimization process. Briefly, we counted low-quality markers for each SLAF marker and for each individual and deleted the worst markers or individuals. We repeated this process, deleting one individual or marker each time. We ceased when the average genotyping quality score of all SLAF markers reached the cutoff value, which was 13.

## Results

### SLAF-seq assay design and pilot studies on rice and soybean

**Figure 2:**
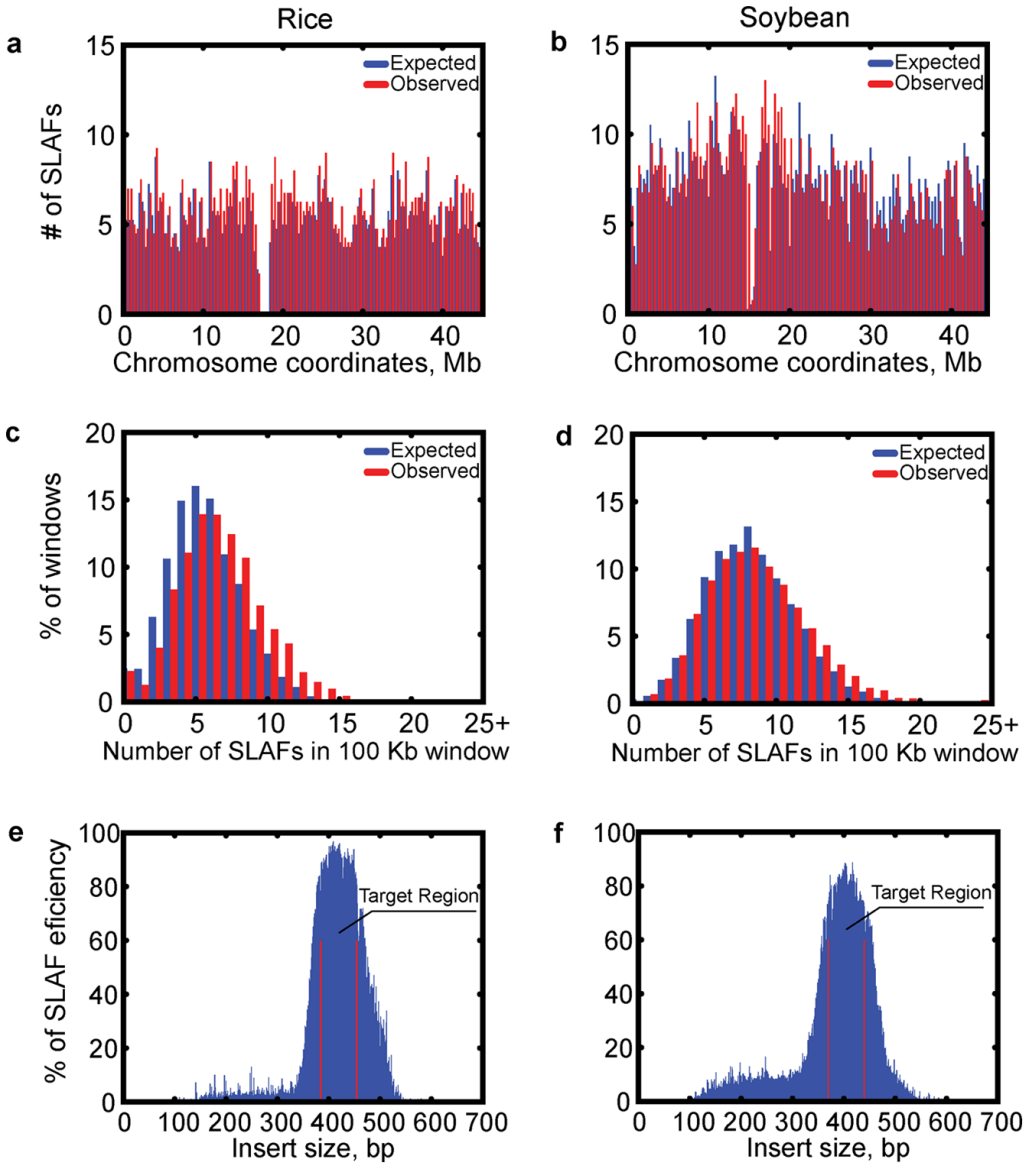
Pilot SLAF-seq data analysis using rice and soybeans. (a)and(b) Insert size distribution of SLAFs. SLAF length was found to cluster tightly around a mean of 430 bp, with 85% of SLAFs in the centermost 50 bp. (c) and (d)Distribution of SLAFs on the chromosomes. SLAFs were evenly distributed on the chromosomes in rice and soybeans. The gap in the middle was caused by the absence of centromere sequences. (e)and(f) Customized SLAF density design. In the rice pilot case, the density was designed using 20 kb per SLAF. In soybeans, 40 kb per SLAF was used. Both rice and soybean pilot SLAF data were found to be consistent with theoretical predictions.

To determine the reliability of this method, we performed a pilot study on rice and soybeans. Specifically, we expected that 21,000 and 76,000 SLAFs would be selected by *Hae*III and *Mse*I digestion and purification restriction fragments. We expected these SLAFs to be about 380∼450 bp and 370∼440 bp for rice and soybeans, respectively, and to comprise about 0.33% and 0.49% of the rice and soybean genomes [5], [6] (Table 1). Repetitive sequences were controlled within 17.78% and 25.32%, respectively, to exclude most of the repetitive regions in rice and soybeans. Using these schemes, we carried out SLAF library construction and sequencing. In total, 0.5 M and 1.3 M pair-end reads were generated using Illumina Genome Analyzer IIx for rice and soybeans, respectively, by mapping the reads to genomes. About 25,000 and 83,000 SLAFs were observed. The SLAF density and distribution characteristics are shown in Figure 2. The properties of the observed SLAFs were consistent with those predicted for both rice and soybeans. In rice, about 90.18% of the expected SLAFs were present in the observed data, which covered 82.48% of the total reads. In soybeans, 79.32% of the expected SLAFs were observed and 65.86% of the total reads were mapped to the expected SLAFs (Table 1). The average SLAF sequence depth was increased by 19-fold and 12-fold to ensure the base qualities. Repetitive SLAFs were controlled within 19.49% and 25.34%, which were consistent with the expected number. The pilot data indicated that the reliability and quality of this SLAF-seq strategy were relatively high.

**Table 1:** Pilot SLAF-seq data summary in rice and soybeans.

Genome information	Rice	Soybeans
Genome size (Mb)	382.79	950.07
% of repeats in genome	39.11%	42.96%
GC content	43.56%	34.67%
**Expected information**		
Enzymes	HaeIII+MseI	HaeIII+MseI
Expected SLAF size range (bp)	384–454	369–439
Expected no. of SLAFs	21,074	76,970
SLAF density per 100 kb	5.51	8.10
Simplification ratio	0.33%	0.49%
% of SLAFs in repeats	17.78%	25.32%
**Observed information**		
Total reads	546,271	1,341,599
Observed no. of SLAFs	25,433	83,055
Matched no. of SLAFs	19,005	61,056
Matched % of SLAFs	90.18%	79.32%
Reads % in matched SLAFs	82.48%	65.86%
SLAF average depth	18.95	12.11
SLAFs per 100 kb	6.64	8.74
Simplification ratio	0.40%	0.52%
% of SLAFs in repeats	19.49%	25.34%

### Large-scale genotyping in common carp F1 population using SLAF-seq

We genotyped a common carp(*Cyprinuscarpio L.*) F1 population *de novo* using SLAF-seq. *Mse*I and *Alu*I were used for library construction. Fragment length was between 330 bp and 380 bp. About 65,000 SLAFS were predicted from the carp genome based on analysis of zebrafish training data. The expected SLAFs may be randomly distributed throughout the zebrafish chromosomes (Figure S2) and the expected repetitive SLAFs may be controlled within 21.3%.

In total, 103.8 M pair-end reads were generated using an Illumina Genome Analyzer IIx for 2 parents and 211progeny simultaneously. On average, 0.4 M reads were obtained for each descendant (Table S1). Based on the sequence similarity, reads were clustered into SLAFs (described in methods). As shown in Table 2, after about 26.02 M reads in low-depth SLAFs and 29.59 M reads in repeat-suspicious SLAFs were excluded, 50,530 high-quality SLAFs were defined with 48.19 M reads. The average sequence depth of these SLAFs was 954-fold with 52.37-fold in parents and 4.99-fold in each individual. The extreme depth of the locus-specific sequences indicates the accuracy of *de novo* SNP discovery.

**Table 2:** SLAF-seq data summary for common carp F1 population.

**Total reads**	
No. of reads	103,800,295
Reads in high quality SLAFs	48,192,694
Reads in repeated SLAFs	29,591,938
Reads in low depth SLAFs	26,015,663
**High quality SLAFs**	
No. SLAFs	50,530
Average SLAF depth	954
Average depth in parents	52.37
Average depth in individuals	4.99
**Polymorphic SLAFs**	
No. of polymorphic SLAFs	10,662
Average depth in parents	57.33
Average. depth in individuals	4.28
No. of SNPs	13,291
No. of InDels	1,483
SNP ratio per kb	4.38
InDel ratio per kb	0.49

A total of 10,662 SLAFs were detected. They contained polymorphisms among which 13,291 SNPs and 1,483InDels were identified. On average, the sequences of parents were 57.33-fold and those of progeny were 4.28-fold. The SNP ratio was about 4.38 per kb, and the InDel ratio was 0.49 per kb in common carp. Based on the barcode system, all polymorphic loci were genotyped separately for parents and 211progeny.

**Figure 3:**
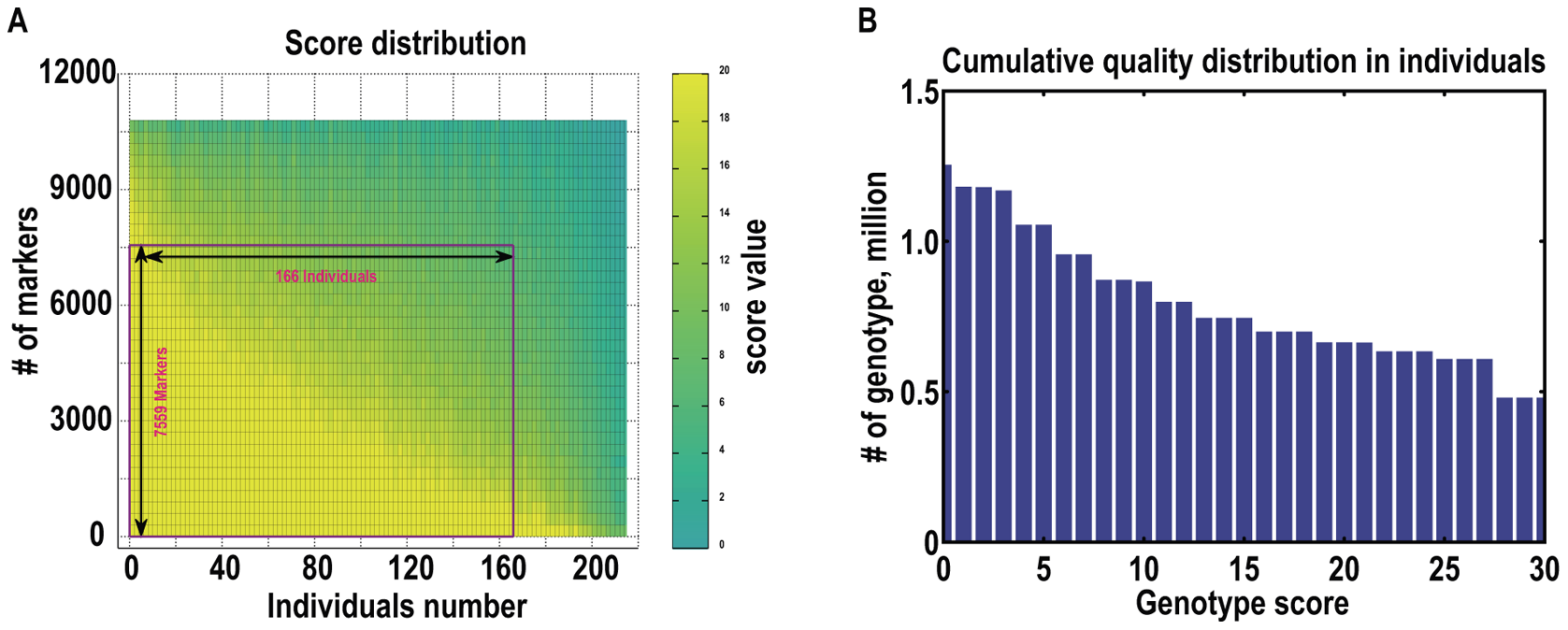
Genotyping quality in common F1 population. The genotyping quality score was used to select qualified markers and individuals for subsequent analysis. This is a dynamic optimization process. We counted low-quality markers for each SLAF marker and for each individual, and deleted the worst marker or individual. We repeated this process, deleting an individual or a marker each time until the average genotyping quality score of all SLAF markers reached the cutoff value, which was 13. (a)Detailed genotyping quality of SLAF-seqdata. (b) Cumulative quality score distribution of 7559 markers in 166 individuals.

Sequencing depth alone cannot fully reflect the genotyping quality. Genotyping quality scores (see methods) were used to evaluate genotyping quality in populations, as shown in Figure 3A. The quality scores used in this approach were transformed from error rates using the expression in PHRED, but error rates were calculated using a new method. Using quality scores, low-quality individuals and low quality markers were excluded, and 7,559 markers in 166 individuals were shown to be high-quality. For genotyping quality in parents, 99.67% of SLAF loci showed genotyping quality scores over 20 with average sequence depth of 68.8-fold. Among individuals, 69% had genotyping scores over 10 with an average sequence depth of 6.2-fold (Figure 3B). To further evaluate the accuracy of genotyping data, we randomly chose 10 SLAF loci in 2 parents and 19 individuals and performed independent traditional Sanger sequencing. Of these 205 genotypes were found to be consistent with the SLAF-seq genotyping information. Five cases of incorrect genotyping were indicated by low genotyping quality scores. Details are shown in Table S2. These types of quality validation all confirmed the genotyping accuracy of SLAF-seq.

### High density and quality genetic map of common carp produced using SLAF-seq genotyping data

A high density genetic map was constructed using the SLAF-seq genotyping data. All markers were grouped in 50 linkage groups with LOD thresholds ranging from 4∼7. Of these, 5,885 markers were arranged on the map using JoinMap4.0 [24]. A male map with 2,158 markers and a female map with 3,106 markers were also generated. The total genetic distance of the sex-averaged map was 3,960 cM. The average interval between markers was 0.68 cM, making this the highest density genetic map yet created for any organism lacking reference genome sequences [25]–[29]. Detailed statistical information for all linkage groups is shown in Table S3 and Figure S3.

**Figure 4:**
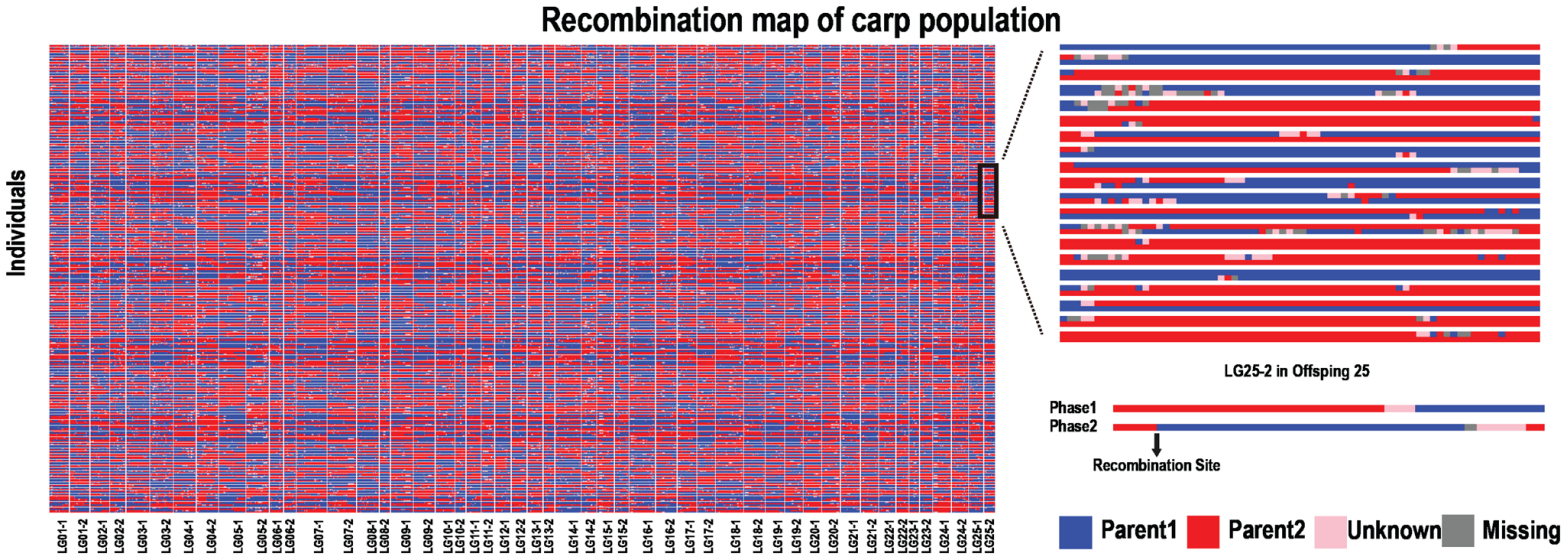
Genetic map validation by recombination mapping. Each two rows represent a genome in a CP population including 211 progenies and 2 parents. Columns correspond to chromosomes. Red and blue shading indicate maternal or paternal haplotype, respectively. Pink shading indicates ambiguous haplotypes, and grey shading indicates missing data. Only 1.51% of the markers were found in small recombination blocks.

**Figure 5:**
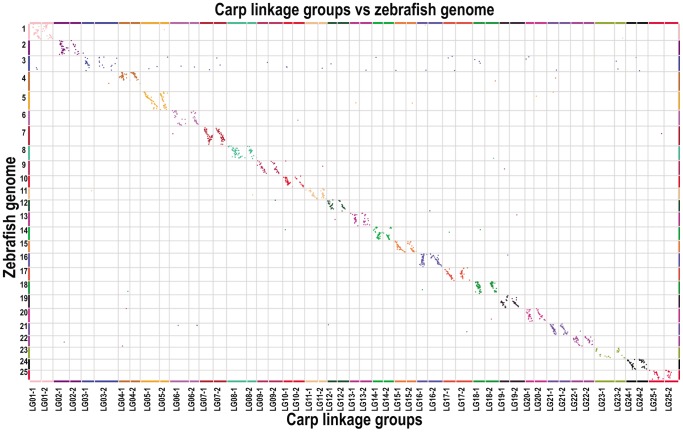
Comparative study between common carp linkage map and zebrafish chromosomes. Two common carp linkage groups corresponded to one zebrafish chromosome.

To evaluate the accuracy of the genetic map, the recombination breakpoints were analyzed to identify questionable genotyping data. Most of the recombination blocks were clearly defined and only 1.51% of the genotyping data were found in very small, doubtful recombination blocks (Figure 4). We mapped SLAF markers to the zebrafish genome. About 25.6% of these markers were uniquely mapped, and 2 carp linkage groups were located in one zebrafish chromosome (Figure 5). The linkage distance was well consistent with the zebrafish physical distance. These two independent approaches all indicated that the marker positions on the map were valid. They also indicated the accuracy of the genotyping results.

## Discussion

The SLAF-seq strategy combines locus-specific amplification and high-throughput sequencing to effect *de novo* SNP discovery and large-scale genotyping. To improve the efficiency of fragment selection, a bioinformatics statistical model was developed for the amplification of specific loci fragments selected based on training data. These included randomly sequenced BAC sequences and genome draft sequences. The training data were taken from both the target organism and from other, evolutionarily related organisms, even from organisms with GC content similar to that of the target organism. A SLAF-efficient selection scheme can then be developed using this training data (Figure 1). We analyzed several training data sets, as shown in Table S4. The repetitive sequences (both mathematically-defined repeat and biologically-defined repeat) can be avoided efficiently and the scheme provided us with a clear set of expectations suitable to the specific purpose of our research project, which improved the RRL efficiency more than in previous studies [18], [20], [22], [29].

To maintain copy number uniformity among fragments, we performed several optimization procedures for amplification and size selection. During the PCR amplification process, the copy number frequency of fragments of different lengths often changed with amplification efficiency (Figure 1). In previous studies, researchers tended to select relatively long length ranges to identify as many enzyme sites as possible because the pre-design step is ignored, allowing the selection of fragments with different copy numbers. This reduces the sequence efficiency [22], [29]. The selection of a tighter length range (about 30∼50 bp) may help to obtain fragments with similar copy numbers and to ensure similar sequence depths among fragments to solve this problem. The amount of template and number of PCR cycles were also optimized to prevent non-specific amplifications and to reduce the frequency of changes in copy number between fragments with similar lengths. This renders research more cost-effective. Most other sequence-based genotyping methods cannot do this [18], [22].

A double barcode system was developed to distinguish individuals in large populations. Two rounds of PCR were performed to add two barcode oligos to each sample (Figure 1). In this study of 211 common carp progeny, 50 individuals were pooled together and tagged with a first-class barcode. Then five groups were pooled together and distinguished with a second-class barcode. A total of only 55 barcode adapters were used to distinguish all these 211 individuals. We developed 96×12 index combinations, which expanded the pool scale more efficiently than the previous single barcode system, making it possible for us to genotype about 10,000 samples in a single high-throughput sequencing run and to reduce the complexity and cost of sample preparation [14]–[16], [18], [30].We used a pair-end sequencing procedure. This allowed us to design primers using the ends of the fragment and to perform further experimental validation, which is especially important for species for which reference genome sequences are not available.

In the present study, the quality score algorithm was developed to evaluate the quality of SNP discovery and genotyping. This quality score can be used to exclude suspicious genotyping results and to improve the accuracy of association studies. This score is suitable for use as a quality standard and can help researchers balance accuracy and costs during heterozygote detection using high-throughput sequencing technology.

Sequencing depth is an important consideration in sequencing-based genotyping. To estimate the minimal sequencing depth required for accurate genotyping results, we randomly simulated genotype subsets of a CP population with various sequencing depths using the Poison process, which was used to simulate the human X chromosome dataset by Bentley et al. [31]. In the present study, the genotype subsets included 200 individuals and 500 markers. The genotyping errors in simulation analysis only include those errors that occurred when one of the two alleles in a heterozygous individual was not successfully sampled and sequenced because of the randomness of sampling. In such cases, a heterozygote would be wrongly called a homozygote. The simulation analysis showed that the error ratio of genotype calling dropped greatly from 1× to 4×, and that further increases in sequencing depth above 4× sequencing depth had relatively little influence on sequencing error rates (Figure S4), relative to the depth changes from 1× to 4×. During genotyping calling, sequencing reads with low depth were filtered away using the quality score, defined by a Bayesian approach. The final sequencing depth of the reads, which was used to generate the genotype data, was greater than the initial average sequencing depth. After balancing cost and quality, we chose to sequence individuals with 4.28× coverage. After filtering out low-depth data, the sequencing depth of the reads that actually generated the final genotype data reached 6.2×. Based on the simulation study of a CP (F1) population (Figure S4), raw data of 6.2× sequencing depth were estimated and used to produce a genotype calling error rate of only 6.5%. This estimation was greater than that discussed in the report by Bentley et al. (about 5.6%) [31]. Our data simulated a CP population, and CP populations have high rates of heterozygosity, which renders genotyping prone to error. The error rate should be smaller than 6.5% for most populations, such as inbred populations. We randomly selected 10 SLAF and validated their accuracy in 19 randomly chosen individuals using Sanger sequencing. We found only five inconsistencies in 190 SLAF data. Further analysis showed that these five SLAF, which were inconsistent with results obtained using Sanger sequencing, had low genotyping quality scores (samples 2, 3, 5, 7, and 9). In subsequent rounds of linkage mapping, we screened incorrect genotypes and deleted some erroneous data. In this way, the study generated a high-quality linkage map, which reflects the accuracy of genotype data. Taken together, weighing the sequencing cost, genotyping quality, and linkage map quality, we concluded that the 6× is an acceptable minimal sequencing depth when this approach is used.

Generally, after the DNA is isolated, there is no difference between genotyping a plant gene and an animal gene. All the procedures of DNA sequencing and of subsequent sequence analysis that apply to plant DNA also apply to animal DNA. However, plant genomes are richer in repetitive sequences than animal genomes. To better understand the efficiency with which the SLAF-seq method filters away repetitive sequences, we used plant genomes to evaluate its reliability. We chose rice because its genome has been sequenced using a BAC-to-BAC sequencing strategy based on the Sanger sequencing platform and because the quality of said genome has been well recognized. However, its genome size is only about 389 Mb. For this reason, we also evaluated the reliability of our method on soybeans, whose 1115 Mb genome is much larger.

Reference genome sequences and reference SNPs were not found to be necessary with this method. *De novo* genotype definition was performed using high-depth sequencing. This overcomes many of the limitations of previous methods [15], [16], [32]. A comparison of different genotyping methods is shown in Table S5. The RRL strategy allows costs to be controlled at the lowest level. Depth sequencing ensures the accurate genotyping. The pre-design strategy can be used to predict fragmentation efficiency reasonably and reliably. The double barcode system allows large populations to be genotyped simultaneously. SLAF-seq combines all these features. SLAF-seq is suitable for widespread use in large-scale genotyping and genome-wide association studies.

## Supporting Information

Figure S1 **Genotype definition process of SLAF-seq.** Four steps were defined to deal with SLAF-seq data. (A) Samples were distinguished by barcodes and datagrouping by sequence similarity. (B) Sequence error evaluation by control data. (C) MAF filtering and SLAF definition. (D) Correction of sequence errors. (E) Definition and evaluation of genotypes.
(PNG)

Figure S2 **Pilot analysis of zebrafish for common carp SLAF pre-design.** (A) SLAF distribution on chromosomes. (B) A distribution of SLAF density in 400 Kb windows. Both A and B indicate a uniformity of distribution.
(TIF)

Figure S3 **Highest density genetic map yet created for organisms without reference genome sequences using common carp F1 population.** 5,885 markers were distributed in 50 linkage groups. Total 3,960 cM was covered with 0.68 cM average intervals.
(PNG)

Figure S4 **Effects of sequencing depth on genotype calling quality.** Simulated genotype subsets with various sequencing depths,including 100 individuals and 500 markers,were generated randomly using the Poison process.The genotyping errors in simulation analysis only included those errors that occurred when one of the two alleles in a heterozygous individual was not successfully sampled and sequenced because of the randomness of sampling. In such cases, a heterozygote was wrongly called a homozygote.The genotyping missing denoted that both the two alleles in an individual fail to be sampled and sequenced due to sampling randomness.
(TIF)

Table S1 **Number of detailed reads in each individual carp.**
(XLSX)

Table S2 **Independent Sanger sequencing for quality validation of SLAF-seq genotyping.**
(XLSX)

Table S3 **Detail statistics of high density common carp genetic map.**
(XLSX)

Table S4 **SLAF-seq pre-designs for different species.**
(XLSX)

Table S5 **Comparison between SLAF-seq and other genotyping technologies.**
(XLSX)

## References

[1] Jeffreys AJ, Wilson V, Thein SL (1985) Hypervariable ‘minisatellite’ regions in human DNA. Nature 314: 67–73. 3856104 10.1038/314067a0

[2] Vos P, Hogers R, Bleeker M, Reijans M, van de Lee T, et al (1995) AFLP: a new technique for DNA fingerprinting. Nucleic Acids Res 23: 4407–4414. 7501463 10.1093/nar/23.21.4407 PMC307397

[3] Williams JG, Kubelik AR, Livak KJ, Rafalski JA, Tingey SV (1990) DNA polymorphisms amplified by arbitrary primers are useful as genetic markers. Nucleic Acids Res 18: 6531–6535. 1979162 10.1093/nar/18.22.6531 PMC332606

[4] Waterston RH, Lindblad-Toh K, Birney E, Rogers J, Abril JF, et al (2002) Initial sequencing and comparative analysis of the mouse genome. Nature 420: 520–562. 12466850 10.1038/nature01262

[5] International Rice Genome Sequencing Project (2005) The map-based sequence of the rice genome. Nature 436: 793–800. 16100779 10.1038/nature03895

[6] Schmutz J, Cannon SB, Schlueter J, Ma J, Mitros T, et al (2010) Genome sequence of the palaeopolyploid soybean. Nature 463: 178–183. 20075913 10.1038/nature08670

[7] Vonholdt BM, Pollinger JP, Lohmueller KE, Han E, Parker HG, et al (2010) Genome-wide SNP and haplotype analyses reveal a rich history underlying dog domestication. Nature 464: 898–902. 20237475 10.1038/nature08837 PMC3494089

[8] Gunderson KL, Steemers FJ, Lee G, Mendoza LG, Chee MS (2005) A genome-wide scalable SNP genotyping assay using microarray technology. Nat Genet 37: 549–554. 15838508 10.1038/ng1547

[9] Atwell S, Huang YS, Vilhjalmsson BJ, Willems G, Horton M, et al (2010) Genome-wide association study of 107 phenotypes in Arabidopsis thaliana inbred lines. Nature 465: 627–631. 20336072 10.1038/nature08800 PMC3023908

[10] Shifman S, Bell JT, Copley RR, Taylor MS, Williams RW, et al (2006) A high-resolution single nucleotide polymorphism genetic map of the mouse genome. PLoS Biol 4: e395. 17105354 10.1371/journal.pbio.0040395 PMC1635748

[11] Xia Q, Guo Y, Zhang Z, Li D, Xuan Z, et al (2009) Complete resequencing of 40 genomes reveals domestication events and genes in silkworm (Bombyx). Science 326: 433–436. 19713493 10.1126/science.1176620 PMC3951477

[12] Rubin CJ, Zody MC, Eriksson J, Meadows JR, Sherwood E, et al (2010) Whole-genome resequencing reveals loci under selection during chicken domestication. Nature 464: 587–591. 20220755 10.1038/nature08832

[13] Lam HM, Xu X, Liu X, Chen W, Yang G, et al (2010) Resequencing of 31 wild and cultivated soybean genomes identifies patterns of genetic diversity and selection. Nat Genet 42: 1053–1059. 21076406 10.1038/ng.715

[14] Xu X, Liu X, Ge S, Jensen JD, Hu F, et al (2012) Resequencing 50 accessions of cultivated and wild rice yields markers for identifying agronomically important genes. Nat Biotechnol 30: 105–111. 10.1038/nbt.2050 22158310

[15] Huang X, Feng Q, Qian Q, Zhao Q, Wang L, et al (2009) High-throughput genotyping by whole-genome resequencing. Genome Res 19: 1068–1076. 19420380 10.1101/gr.089516.108 PMC2694477

[16] Xie W, Feng Q, Yu H, Huang X, Zhao Q, et al (2010) Parent-independent genotyping for constructing an ultrahigh-density linkage map based on population sequencing. Proc Natl Acad Sci U S A 107: 10578–10583. 20498060 10.1073/pnas.1005931107 PMC2890813

[17] Altshuler D, Pollara VJ, Cowles CR, Van Etten WJ, Baldwin J, et al (2000) An SNP map of the human genome generated by reduced representation shotgun sequencing. Nature 407: 513–516. 11029002 10.1038/35035083

[18] Hyten DL, Cannon SB, Song Q, Weeks N, Fickus EW, et al (2010) High-throughput SNP discovery through deep resequencing of a reduced representation library to anchor and orient scaffolds in the soybean whole genome sequence. BMC Genomics 11: 38. 20078886 10.1186/1471-2164-11-38 PMC2817691

[19] Lucito R, Nakimura M, West JA, Han Y, Chin K, et al (1998) Genetic analysis using genomic representations. Proc Natl Acad Sci U S A 95: 4487–4492. 9539764 10.1073/pnas.95.8.4487 PMC22516

[20] Van Tassell CP, Smith TP, Matukumalli LK, Taylor JF, Schnabel RD, et al (2008) SNP discovery and allele frequency estimation by deep sequencing of reduced representation libraries. Nat Methods 5: 247–252. 18297082 10.1038/nmeth.1185

[21] Sachidanandam R, Weissman D, Schmidt SC, Kakol JM, Stein LD, et al (2001) A map of human genome sequence variation containing 1.42 million single nucleotide polymorphisms. Nature 409: 928–933. 11237013 10.1038/35057149

[22] Baird NA, Etter PD, Atwood TS, Currey MC, Shiver AL, et al (2008) Rapid SNP discovery and genetic mapping using sequenced RAD markers. PLoS One 3: e3376. 18852878 10.1371/journal.pone.0003376 PMC2557064

[23] Kent WJ (2002) BLAT–the BLAST-like alignment tool. Genome Res 12: 656–664. 11932250 10.1101/gr.229202 PMC187518

[24] Van Ooijen JW (2006) JoinMap 4, Software for the calculation of genetic linkage maps in experimental populations. Kyazma BV, Wageningen, Netherlands.

[25] Graham IA, Besser K, Blumer S, Branigan CA, Czechowski T, et al (2010) The genetic map of Artemisia annua L. identifies loci affecting yield of the antimalarial drug artemisinin. Science 327: 328–331. 20075252 10.1126/science.1182612

[26] Bindler G, Plieske J, Bakaher N, Gunduz I, Ivanov N, et al (2011) A high density genetic map of tobacco (Nicotiana tabacum L.) obtained from large scale microsatellite marker development. Theor Appl Genet 123: 219–230. 21461649 10.1007/s00122-011-1578-8 PMC3114088

[27] Somers DJ, Isaac P, Edwards K (2004) A high-density microsatellite consensus map for bread wheat (Triticum aestivum L.). Theor Appl Genet 109: 1105–1114. 15490101 10.1007/s00122-004-1740-7

[28] Wenzl P, Carling J, Kudrna D, Jaccoud D, Huttner E, et al (2004) Diversity Arrays Technology (DArT) for whole-genome profiling of barley. Proc Natl Acad Sci U S A 101: 9915–9920. 15192146 10.1073/pnas.0401076101 PMC470773

[29] Amores A, Catchen J, Ferrara A, Fontenot Q, Postlethwait JH (2011) Genome evolution and meiotic maps by massively parallel DNA sequencing: spotted gar, an outgroup for the teleost genome duplication. Genetics 188: 799–808. 21828280 10.1534/genetics.111.127324 PMC3176089

[30] Binladen J, Gilbert MT, Bollback JP, Panitz F, Bendixen C, et al (2007) The use of coded PCR primers enables high-throughput sequencing of multiple homolog amplification products by 454 parallel sequencing. PLoS One 2: e197. 17299583 10.1371/journal.pone.0000197 PMC1797623

[31] Bentley DR, Balasubramanian S, Swerdlow HP, Smith GP, Milton J, et al (2008) Accurate whole human genome sequencing using reversible terminator chemistry. Nature 456: 53–59. 18987734 10.1038/nature07517 PMC2581791

[32] Kennedy GC, Matsuzaki H, Dong S, Liu WM, Huang J, et al (2003) Large-scale genotyping of complex DNA. Nat Biotechnol 21: 1233–1237. 12960966 10.1038/nbt869

