# Supplementary figures and images for: SLAF-seq: An Efficient Method of Large-Scale De Novo SNP Discovery and Genotyping Using High-Throughput Sequencing
Source: PLoS One. 2013 Mar 19;8(3):e58700. doi: 10.1371/journal.pone.0058700 (PMC3602454; doi:10.1371/journal.pone.0058700)

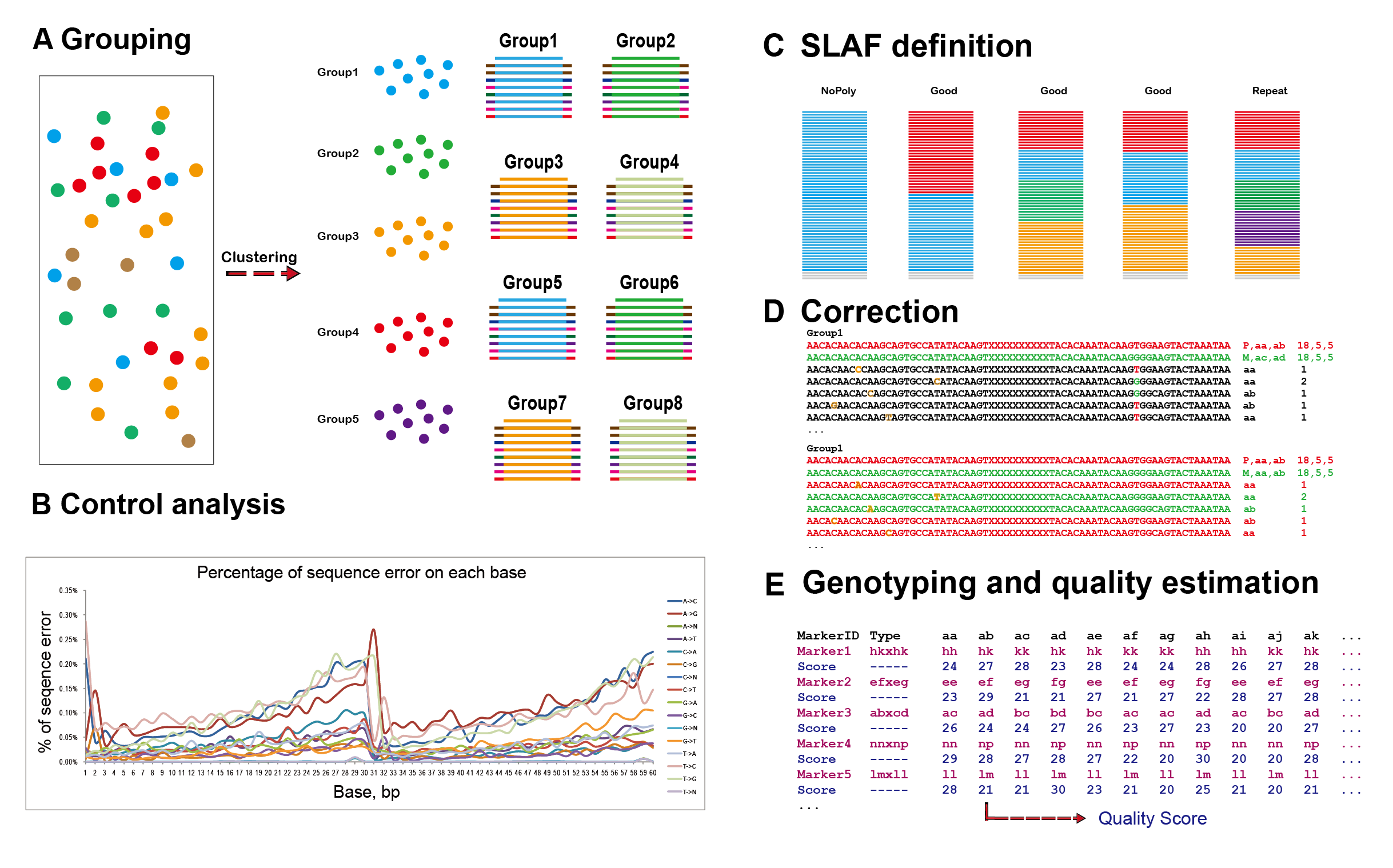

Supplement: Figure S1 — Genotype definition process of SLAF-seq. Four steps were defined to deal with SLAF-seq data. (A) Samples were distinguished by barcodes and datagrouping by sequence similarity. (B) Sequence error evaluation by control data. (C) MAF filtering and SLAF definition. (D) Correction of sequence errors. (E) Definition and evaluation of genotypes. (PNG) [file pone.0058700.s001.png]

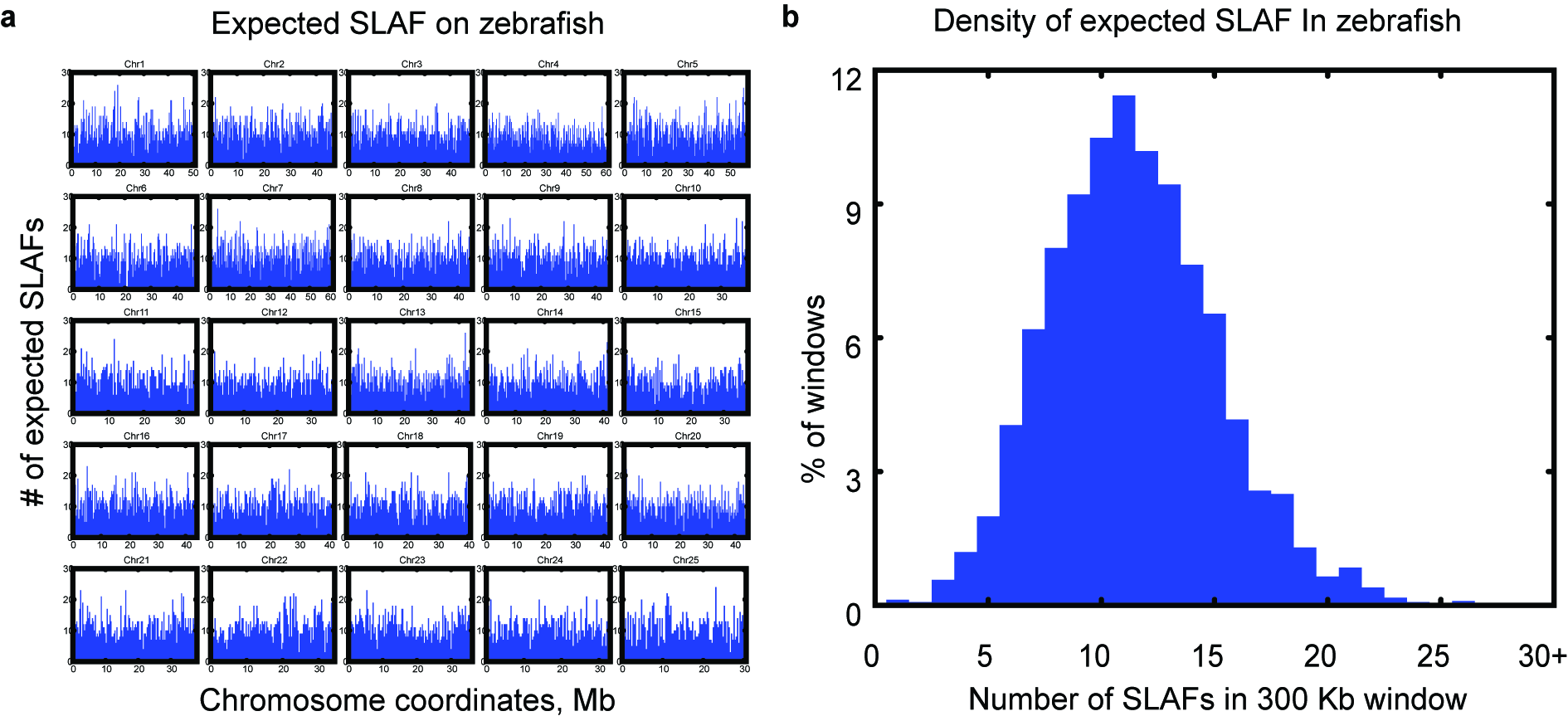

Supplement: Figure S2 — Pilot analysis of zebrafish for common carp SLAF pre-design. (A) SLAF distribution on chromosomes. (B) A distribution of SLAF density in 400 Kb windows. Both A and B indicate a uniformity of distribution. (TIF) [file pone.0058700.s002.tif]

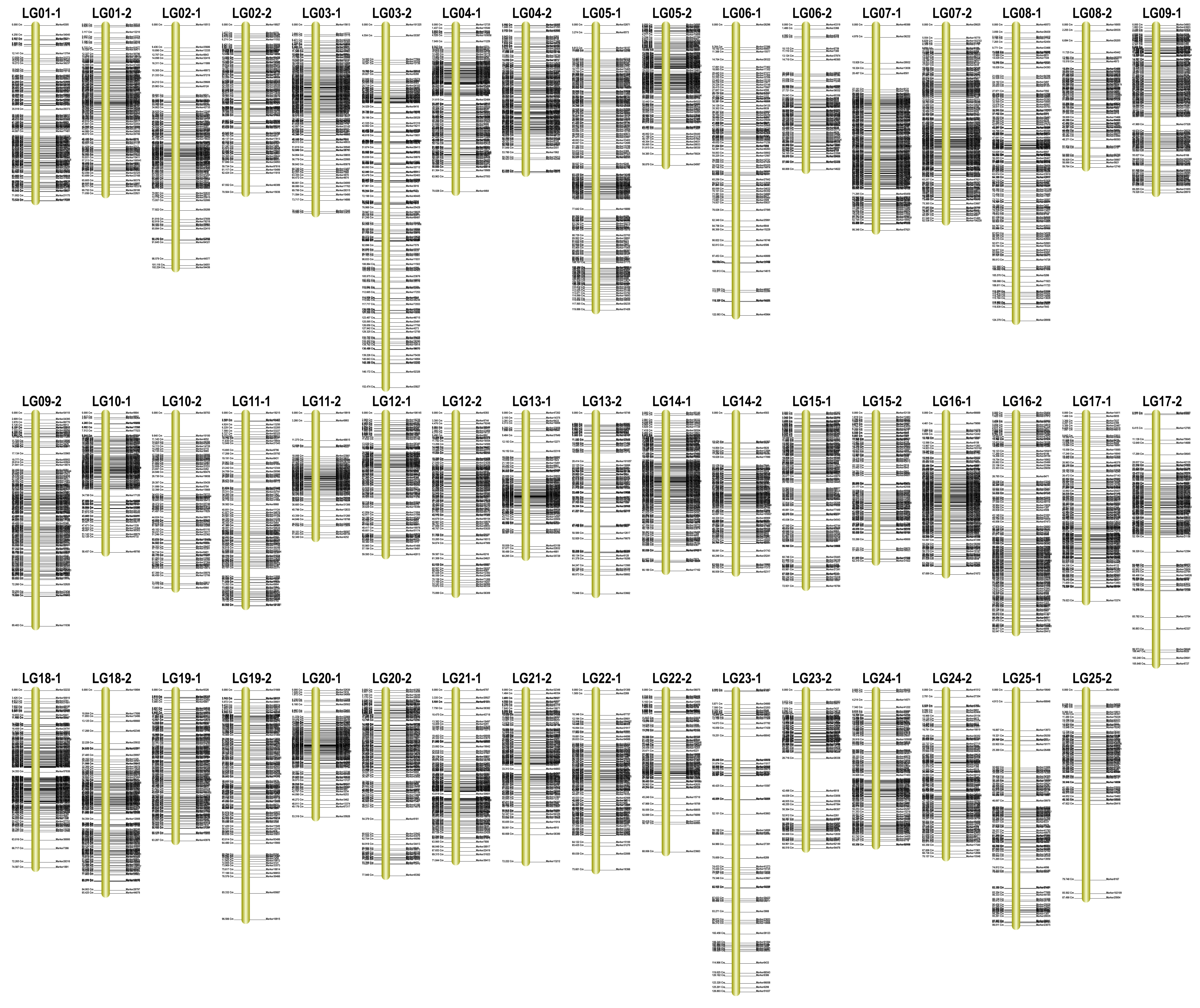

Supplement: Figure S3 — Highest density genetic map yet created for organisms without reference genome sequences using common carp F1 population. 5,885 markers were distributed in 50 linkage groups. Total 3,960 cM was covered with 0.68 cM average intervals. (PNG) [file pone.0058700.s003.png]

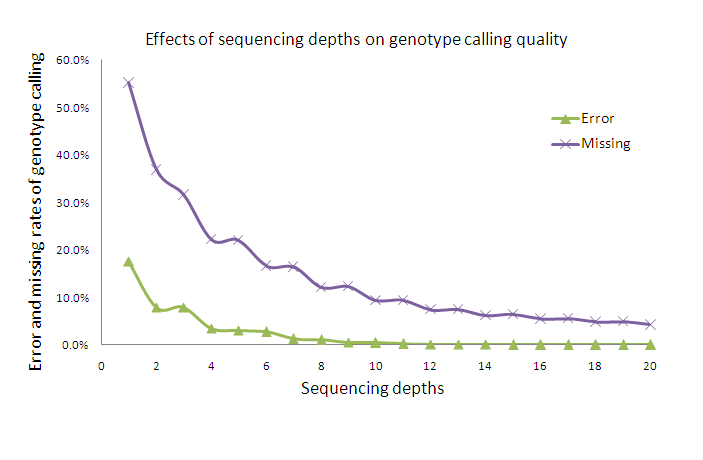

Supplement: Figure S4 — Effects of sequencing depth on genotype calling quality. Simulated genotype subsets with various sequencing depths,including 100 individuals and 500 markers,were generated randomly using the Poison process.The genotyping errors in simulation analysis only included those errors that occurred when one of the two alleles in a heterozygous individual was not successfully sampled and sequenced because of the randomness of sampling. In such cases, a heterozygote was wrongly called a homozygote.The genotyping missing denoted that both the two alleles in an individual fail to be sampled and sequenced due to sampling randomness. (TIF) [file pone.0058700.s004.tif]
